# Response: Commentary: Measuring Counterintuitiveness in Supernatural Agent Dream Imagery

**DOI:** 10.3389/fpsyg.2020.00221

**Published:** 2020-02-26

**Authors:** Andreas Nordin, Pär Bjälkebring

**Affiliations:** ^1^Department of Cultural Sciences, University of Gothenburg, Gothenburg, Sweden; ^2^Department of Psychology, University of Gothenburg, Gothenburg, Sweden

**Keywords:** dreaming, cognition, counterintuition, supernatural agent concept, religion, CI scheme

(Sears, 2019) comment partly addresses the main purpose of our article (Nordin and Bjälkebring, 2019) about counterintuitiveness in dreaming. While we recognize that his remarks include some constructive points about the presented research results and highlight some possible limitations in the theoretical summary and modeling of supernatural agent (SA) cognition in dreaming, we take issue with other points and with the way our arguments are framed. In this response, we suggest that, given the prominent research in the field, parts of Sears's comment are overly dismissive and neglect to take into account key aspects of our specific arguments and our modeling of SA cognition in dreaming.

## Sears on Counterintuitive Objects in Dreams

Sears offers a brief and partly constructive depiction of the empirical study described in the article. Some of our main arguments and aims, however, despite comprising the most significant part of the contribution, are omitted or only vaguely discussed. These include to measure the general pervasiveness of counterintuitiveness, to test (Barrett, 2008) counterintuitiveness coding and quantifying scheme in the context of religious dreaming by assessing intercoder reliability, and to explore the prevalence and base-rate frequency of counterintuitiveness in dream reports, and thereby establish cross-cultural base rates of counterintuitiveness in dreams for future research.

We agree with Sears's comments on our results about supernatural artifacts in dreams, on “cultural influences,” and on the continuity hypothesis [see, e.g., (Domhoff, 1996; Bulkeley, 2009)]. The dreams undoubtedly draw material from and make reference to daytime experience and take cues from the cultural environment, which in the context of the present study includes Hindu imagery, “iconography,” local worship, mythology, and visual culture. Sears's comment leads to welcome and constructive suggestions for future (cross-cultural) research: for instance, the prediction that a Christian or Muslim sample should reveal a lower frequency of counterintuitive artifacts than a Hindu one.

## Sears on Our Model of Supernatural Agent Cognition

In the article, we refer to various studies (McNamara and Bulkeley, 2015; McNamara, 2016; McNamara et al., 2018) that stress the prevalence of co-occurrence of a diminished sense of personal agency and a tendency to construct SA cognition during rapid eye movement (REM) sleep. One possible explanation of this that we discuss is the hypothesis that dreamers produce SA cognition about agency in searching for extrinsic event causes during REM sleep (references above). We share Sears's questions about why this would be the case, and future research will probably offer a more complete answer. As we state in the article, there are strong *prima facie* reasons to suggest that, in this dream context, the ascription of agency, agent causality, and mentality is partly due to a proclivity to adopt a Theory of Mind (ToM), and that this occurs in a manner theorized as hypersensitive agent detection (HADD). We also discuss various “mind prediction” models (p. 6). We too think it remains unclear why, as McNamara et al. (2018) put it, “anyone would postulate SAs in the first place” given these processes, and even more to the point, why SAs should be counterintuitive.

Sears holds that our article employs important yet vaguely defined concepts such as “threat,” “anxiety,” and “strategic information.” The importance Sears imputes to these concepts is somewhat exaggerated, particularly in relation to the limited space of the given article. Contrary to Sears's claim, the notion of “strategic information” is in fact described on page 7 and that of “threat” on page 5.

Furthermore, “threat” and “anxiety” were mentioned due to their functions in some of the prominent models to which we refer as theoretical background conditions for the generation of SA dreams. The empirical aims of the article, however, were (a) to map the prevalence of counterintuitiveness in dreaming, given how much attention minimal counterintuitive (MCI) theory has earned in the scholarly debate and (b) to test (Barrett, 2008) counterintuitiveness coding system. The aim was not to test any functions of “threat” and “anxiety” in the production of SA dreaming. Despite this, Sears contends that we are unable to demonstrate that dreamers experience threat and anxiety prior to the appearance of SAs. This seems to us like an irrelevant objection if it is meant as an attempt to refute our arguments and results. It also goes further astray by suggesting that we should adopt another theoretical framework altogether (seemingly Sears's own). We certainly welcome new explanatory theories if they are relevant and demonstrate parsimony. However, we are not unaware of, much less do we ignore, as Sears seems to imply, the notion of unexpectedness and the kinds of concepts he obviously favors and advocates. Nor do we dispute the viability of unexpectedness as a scientific concept derived from various “mind prediction” approaches; in fact, we discuss precisely these processes on page 6 in the article. We find it not altogether relevant, fair, or reasonable to dismiss our arguments and results about counterintuitiveness in dreaming on the basis that some other concept ought to have been used instead.

In sum, Sears's remarks offer some constructive suggestions and discussion, but we take issue with the overly dismissive comments on the article. We consider the criticism of vagueness to be exaggerated, while the criticism that we lack evidence of threat/anxiety in the empirical data is simply irrelevant, given the aim of the article. The charge of the article's limited value also has low credibility, even from the commenter's point of view, because (a) references to our most important concepts and to the entire research field of MCI and counterintuitiveness are omitted from Sears's criteria; (b) it overestimates *a priori* the explanatory value of other less well-established concepts in the field; and (c) it wrongly suggests that the article lacks any description of “unexpectedness”-type phenomena, when in fact it does describe them. Further, the charge of limited value is arbitrary and self-defeating as it presumes that “sensory gating/deprivation plays important roles in at least some SA dreams”—a statement that both begs the question and invalidates its own claim because its scope is limited by the qualification “in *at least some* SA dreams.” We similarly find the assertion that our study is of limited value because we omitted the commenter's own stance—on “ideal” content situations (Sears, 2016)—to be rather question-begging and even biased.

## Author Contributions

AN authored this response to Dr. Sears's comment on the article Commentary: Measuring Counterintuitiveness in Supernatural Agent Dream Imagery. PB contributed to with comments and suggestions on the response.

### Conflict of Interest

The authors declare that the research was conducted in the absence of any commercial or financial relationships that could be construed as a potential conflict of interest.
